# Epidemiological Scenario of Anisakidosis in Spain Based on Associated Hospitalizations: The Tip of the Iceberg

**DOI:** 10.1093/cid/ciy853

**Published:** 2018-10-03

**Authors:** Zaida Herrador, Álvaro Daschner, María Jesús Perteguer, Agustín Benito

**Affiliations:** 1 National Centre for Tropical Medicine, Instituto de Salud Carlos III, Madrid, Spain; 2 Network of Biomedical Research on Tropical Diseases, Madrid, Spain; 3 Servicio de Alergia, Instituto de Investigación Sanitaria–Hospital Universitario de La Princesa, Madrid, Spain; 4 National Center of Microbiology, Instituto de Salud Carlos III, Madrid, Spain

**Keywords:** anisakidosis, gastroallergic anisakidosis, food parasitology, Anisakis infection, Spain

## Abstract

**Background:**

The risk of infection with *Anisakis* has been recognized for some time, but it is now emerging due to major awareness, better diagnostic techniques, and increasing preference for raw or lightly cooked food. Spain has the second-highest reported incidence after Japan, though the real anisakidosis burden is unknown because of the scarcity of epidemiological data. This study provides a 19-year review of anisakidosis-related hospitalizations describing epidemiological trends and patient characteristics.

**Methods:**

We performed a retrospective descriptive study using the Spanish Hospitalization Minimum Data Set from 1997 to 2015. Hospitalization rates were calculated and spatial distribution of cases and their temporal behavior were assessed. Clinical characteristics were described, including related codiagnoses and procedures.

**Results:**

A total of 2471 hospital discharges were identified. A continuous increasing trend was observed, with several peaks. Most affected communities were located in the northwest inland part of the country. Almost 54% of hospitalized patients were male, with a mean age of 51.3 years. Median length of stay was 5 days, and the hospitalization median cost around €2900. Fatal outcome occurred in 0.5%. Most frequent codiagnoses were digestive diseases, mainly intestinal obstruction. Urticaria, anaphylactic reaction, and angioneurotic edema were only recorded in 2.2%, 2.4%, and 1.2%, respectively.

**Conclusions:**

Knowing that hospitalization is unusual in anisakidosis, we offer calculations of the real disease burden. Improving disease surveillance in parallel to disease control will be useful both in gaining extended disease knowledge and reducing morbidity and related costs.

## Teaser

Anisakidosis is an emerging and underestimated zoonosis in Europe. Spain has the second-highest reported incidence worldwide; however, the real anisakidosis burden remains unknown. We analyzed anisakidosis-related hospitalizations in terms of clinical manifestations and temporal and geographic patterns.

Anisakidosis is a fish-borne zoonosis caused by the ingestion of raw or undercooked fish or cephalopods contaminated by live larvae of parasitic nematodes belonging to the family Anisakidae. This family includes several genera, among which *Anisakis*, *Pseudoterranova*, and *Contracaecum* are more common. The indirect life cycle of anisakids involves various hosts at different levels across the food web, including cetaceans (for *Anisakis*) and pinnipeds (for *Pseudoterranova* and *Contracaecum*) as final hosts, planktonic or semi-planktonic organisms as intermediate hosts, and fish and cephalopods as paratenic hosts [1]. For decades, only cod and herring were considered species at risk (parasitized with *Pseudoterranova* and *Anisakis* species, respectively). To date, we know that most of the marine fish species that reach the markets can be parasitized by different anisakid larvae, mainly *Anisakis* species [2].

Humans are an accidental host in which the worms cannot survive or reproduce. After eating raw or undercooked parasitized marine fish and cephalopods, the parasite penetrates the gastrointestinal tract causing gastrointestinal illness, ectopic reactions, or allergic manifestations [3]. There are 2 different physiopathological mechanisms that justify anisakidosis clinical manifestations. First is the immediate immunoglobulin E (IgE)–mediated hypersensitivity produced by the organism when it recognizes *Anisakis simplex* species antigens released by the larvae as foreign. This mechanism is responsible for acute allergic urticaria, angioedema, or anaphylaxis. The other group of clinical manifestations is due to the local effect of the nematode and the concomitant inflammatory reactions in the segment of the digestive tube where it settles. Depending on the degree of penetration in the mucosa, there are different clinical forms: luminal or noninvasive form (normally asymptomatic), and the invasive form, which in turn can be gastric or intestinal, depending on the affected segment [4]. Extraintestinal locations such as the tongue, pharynx wall, lung, lymphatic ganglia, or pancreas have also been described [5]. All clinical features involve production of specific IgE [6].

Due to the scarcity of epidemiological data, the anisakidosis burden remains unknown [7]. According to the European Food Safety Authority, there were 20000 anisakidosis cases worldwide prior to 2010, with>90% from Japan. Spain appears to have the second-highest reported incidence [8]. In this country, marinated anchovies are recognized as the main food vehicle [9]. The first human case in Spain was reported in the scientific literature in 1991 [10]. To date, there is no surveillance system for anisakidosis in this country. In this article, we aim at describing, for the first time, anisakidosis-related hospitalizations in Spain in terms of time, geographical distribution, and disease characteristics.

## METHODS

### Data Analysis

We performed a retrospective descriptive study using the Hospitalization Minimum Data Set (CMBD in Spanish) for the time period 1 January 1997 to 31 December 2015. The CMBD is the official database of the Spanish Ministry of Health, and collects demographic and clinical information on discharge of all public hospital admissions nationwide. The National Health System (NHS) provides free medical care to 99.5% of the Spanish population, although those persons not covered by the NHS can be attended to at the public hospitals. Since 2005, CMBD also has had a gradual coverage from private hospitals [11]. The *International Classification of Diseases, Ninth Revision, Clinical Modification* (*ICD-9-CM*) was used for this purpose [12]. Registers with an *ICD-9-CM* code of anisakidosis (127.1) placed in any diagnostic position were analyzed.

The average number of anisakidosis hospitalizations per year and for each autonomous community was calculated to assess temporal and geographical patterns. Anisakidosis annual hospitalization rates were computed using the official national and regional population figures at 1 January of every study year. These figures were used as population at risk [13]. Results in terms of mean rates were plotted in maps using the Geographical Information System QGis free software version 2.18.13.

We assessed the trends in anisakidosis-related hospitalizations using linear regression and Joinpoint Poisson regression models (Joinpoint software version 4.2.0.1, National Cancer Institute, Bethesda, Maryland). Temporal trends examining hospitalization rates were generated by fitting log-linear regression models. This technique provides estimates of annual percentage change (APC) in trends with corresponding 95% confidence intervals (CIs). We also estimated the current anisakidosis burden based on the number of cases requiring hospitalization, previous data from the literature, and the calculated time trend for our series.

For each entry, we collected sociodemographic and clinical data. Relevant codiagnoses were explored: We searched for allergic-type codiagnoses as well as digestive codiagnoses to analyze separately classical anisakidosis and gastroallergic anisakidosis. Anisakidosis-related medical/surgical procedures were also analyzed. Frequencies and percentages were used to summarize data. Bivariate associations were assessed using the χ^2^ test. *P* values <.05 were considered statistically significant. Data analysis was performed using Stata software version 14.

### Ethics Statement

This study involves the use of patient medical data from the CMBD. These data are hosted by the Ministry of Health, Consumer Affairs, and Social Welfare. Researchers working in public and private institutions can request the databases by completing, signing, and sending a questionnaire available on the Ministry website. In this questionnaire, a signed confidentiality commitment is required. According to this confidentiality commitment, researchers cannot provide the data to other researchers, who must request the data directly to the Ministry. All data are anonymized and de-identified by the Ministry before being provided to applicants [11].

## RESULTS

### Temporal and Special Trends in Spain

A total of 2471 hospital discharges with a diagnosis of anisakidosis placed in any diagnostic position were identified for the 19-year study period. The mean anisakidosis hospitalization rate was 2.93 per 1000000 population. There was a significant increasing trend in the hospitalization rates during the whole study period (*P* < .05), with 2 peaks in 2002 and 2014. Two significant jointpoints were identified: from 1997 to 2002 (APC, 58.9 [95% CI, 32.8–90]; *P* = .017) and from 2009 to 2015 (APC, 19.6 [95% CI, 4.4–36.9]; *P* = .017).

**Figure 1.**
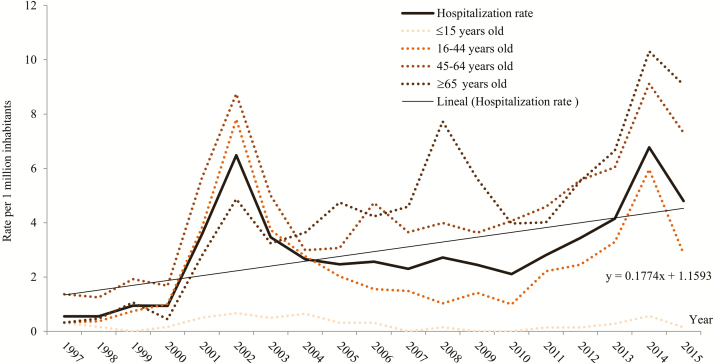
Temporal trend of anisakidosis-related hospitalizations by age group, 1997–2015, Spain.

**Figure 2.**
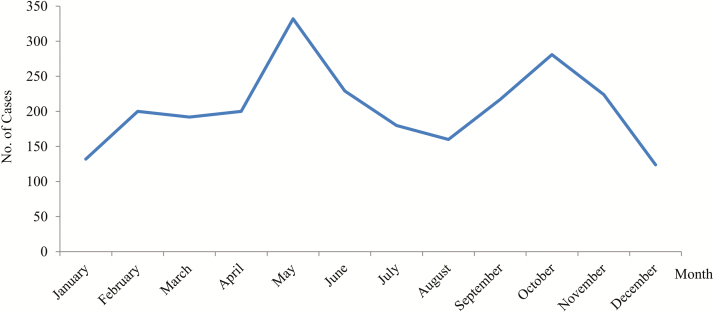
Monthly distribution of anisakidosis-related hospitalizations, 1997–2015, Spain.

Hospitalization rates by age group significantly varied throughout the study period (*P* < .05). The age group 45–64 years showed the highest rate, followed by ages 16–44 years. The 2002 and 2014 peaks were observed in all the age groups, except for those aged ≤15 years (Figure 1). The 13.4% and 11.4% of anisakidosis-related hospitalizations occurred in May and October, respectively (Figure 2).

**Figure 3.**
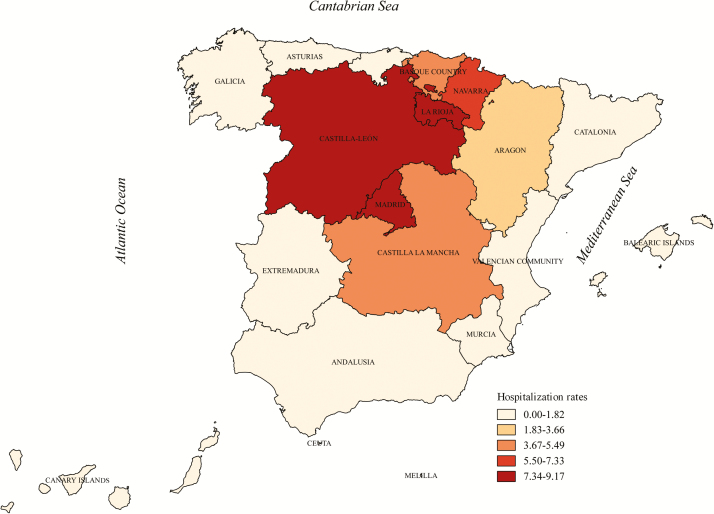
Anisakidosis mean hospitalizations rates per 1000000 population by autonomous community, 1997–2015, Spain.

Regarding the regional distribution, Madrid had the highest mean anisakidosis hospitalization rate (9.17 hospitalizations/1000000 population), followed by Castilla-León (8.99/1000000 population) and La Rioja (8.29/1000000 population). Mean hospitalization rates were <5 hospitalizations/1000000 population in the rest of the autonomous communities (Figure 3; Supplementary Table 1).

### Sociodemographic Characteristics and Clinical Features of Anisakidosis-related Hospitalizations

Among hospitalized patients, 53.6% were male. Mean age was 51.3 years, significantly lower in males than in females (50.5 vs 52.3, respectively; *P* = .011). The median length of stay was 5 days, and the hospitalization median cost was €2922. The length of stay increased with age: 18.8% and 43.3% of patients aged ≤15 and >65 years, respectively, stayed >1 week (*P* < .01). Fatal outcome occurred in 0.5% of all hospitalizations, being more frequent among those aged >65 years (*P* < .01). Six of the 11 deceased patients had some type of neoplasia. The remaining 5 had a history of cardiac and/or pulmonary chronic pathology (Table 1).

**Table 1.** Clinical Characteristics of Anisakidosis Hospitalizations, 1997–2015, Spain

Characteristic	Variable	No. (%)
Sex	Male	1318 (53.3)
	Female	1153 (46.7)
Age group	≤15 y	32 (1.3)
	16–44 y	881 (35.7)
	45–64 y	913 (36.9)
	≥65 y	645 (26.1)
Type of admission	Urgent	2130 (86.2)
	Programmed	340 (13.8)
	Others/unknown	2 (0.1)
Surgical intervention	No	2059 (83.3)
	Yes	412 (16.7)
Type of discharge	Home	2432 (98.4)
	Transfer	10 (0.4)
	Others/unknown	18 (0.7)
	Exitus	11 (0.5)
Readmission	No	2332 (94.4)
	Yes	139 (5.6)
Hospitalization time, d, median (range)		5 (0–176)^a^
Hospitalization cost, median (range)		€2922.6 (€952–€1116848.4)^b^

^a^The patient with 0 days of stay was transferred to another hospital.

^b^The patient with the highest cost underwent surgery due to a hemorrhage of the gastrointestinal tract.

Anisakidosis *ICD-9-CM* code was positioned as the first diagnosis in 47.3% of all related hospitalizations. Other frequent reasons for hospital admission (codiagnoses placed in first position) were mainly diseases of the digestive system, such as intestinal obstruction without mention of hernia (4.9%), other and unspecified noninfectious gastroenteritis and colitis (3.8%), and other symptoms involving abdomen and pelvis (3.2%).

Overall, intestinal obstruction without mention of hernia occurred in 12.6% of all anisakidosis hospitalizations, 8% had other symptoms involving abdomen and pelvis, 3.6% had regional enteritidis, and 3.2% had cholelithiasis. Males and those aged 45–64 years were significantly more prone than females to have developed an intestinal obstruction (*P* < .01). The presence of other and unspecified noninfectious gastroenteritis and colitis, regional enteritis, and other symptoms involving the abdomen and pelvis significantly decreased with age (*P* < .01), while the risk of cholelithiasis increased with age (*P* < .01).

Other frequent codiagnoses were cardiac dysrhythmias (5.6%) and asthma (4.4%), the latter being significantly more common in women than in men (*P* < .01). While cardiac dysrhythmias mainly occurred among the elderly, the percentage of asthma was higher at both age extremes (*P* < .01).

Urticaria, anaphylactic reaction, and angioneurotic edema were recorded in 2.2%, 2.4%, and 1.2% of anisakidosis-related hospitalizations, respectively. There were no sex differences. By age group, we observed that the risk of anaphylactic reaction and angioneurotic edema increased with age (Table 2). Within these 3 groups, concomitant diagnoses of drug allergy accounted for 16.3%, 41.4%, and 26.7% of cases (Supplementary Table 2).

**Table 2.** Percentage of Frequent Codiagnoses in Anisakidosis-related Hospitalizations by Age Group, Spain, 1997–2015

Frequent Codiagnoses	Age Group				*P* Value
	≤15 y	16–44 y	45–64 y	≥65 y	
Most frequent digestive codiagnoses, %					
Intestinal obstruction without mention of hernia	6.25	10.33	17.20	9.61	.000
Gastritis and duodenitis	0.00	7.38	8.87	7.13	.187
Other and unspecified noninfectious gastroenteritis and colitis	18.75	10.22	6.68	4.81	.000
Regional enteritis	6.25	5.22	3.40	1.55	.002
Cholelithiasis	0.00	1.02	3.72	5.58	.000
Most frequent allergy-related codiagnoses, %					
Other anaphylactic reaction. Anaphylactic reaction due to unspecified food	0.00	1.02	2.85	3.88	.002
Urticaria	0.00	1.82	2.52	2.48	.580
Angioneurotic edema, not elsewhere classified	0.00	0.34	1.97	1.24	.014
Other frequent codiagnoses, %					
Other symptoms involving abdomen and pelvis	15.63	10.78	7.45	4.50	.000
Cardiac dysrhythmias	0.00	0.60	3.80	15.20	.000
Asthma	6.25	3.97	3.07	6.67	.006

A subanalysis in patients with allergic symptoms showed 29.1% and 17.2% of digestive codiagnoses in urticaria and angioneurotic edema, respectively, but most importantly only 5% in anaphylactic reactions.

Table 3 summarizes the most frequent medical/surgical procedures. Endoscopy was the most frequent surgical procedure, followed by colonoscopy, gastroscopy, and intestinal partial resection/biopsy. Computed axial tomography and/or diagnostic ultrasound of abdomen were performed in almost one-third of the anisakidosis hospitalizations (Table 3).

**Table 3.** Some of the Most Frequent Procedures in Anisakidosis-related Hospitalizations, Spain, 1997–2015

*ICD-9-CM* Code	Procedures	No.	(%)
Operations on the digestive system			
45.13	Other endoscopy of small intestine	131	(5.3)
45.23	Colonoscopy	124	(5.0)
44.13	Other gastroscopy	122	(4.9)
45.25	Closed [endoscopic] biopsy of large intestine	122	(4.9)
45.62	Other partial resection of small intestine	104	(4.2)
45.16	Esophagogastroduodenoscopy with closed biopsy	104	(4.2)
47.xx	Operations on appendix	88	(3.6)
44.14	Closed [endoscopic] biopsy of stomach	62	(2.5)
45.14	Closed [endoscopic] biopsy of small intestine	38	(1.5)
Miscellaneous diagnostic procedures			
88.01	Computerized axial tomography of abdomen	799	(32.3)
88.76	Diagnostic ultrasound of abdomen and retroperitoneum	797	(32.3)
90.59	Microscopic examination of specimen from musculoskeletal system and of joint fluid, other microscopic examination	360	(14.6)
88.19	Other X-ray of abdomen	356	(14.4)
87.44	Routine chest X-ray, so described	351	(14.2)
Other procedures			
39.96	Total body perfusion	43	(1.7)

Abbreviation: *ICD-9-CM*, International Classification of Diseases, Ninth Revision.

### Anisakidosis Burden in Spain

**Figure 4.**
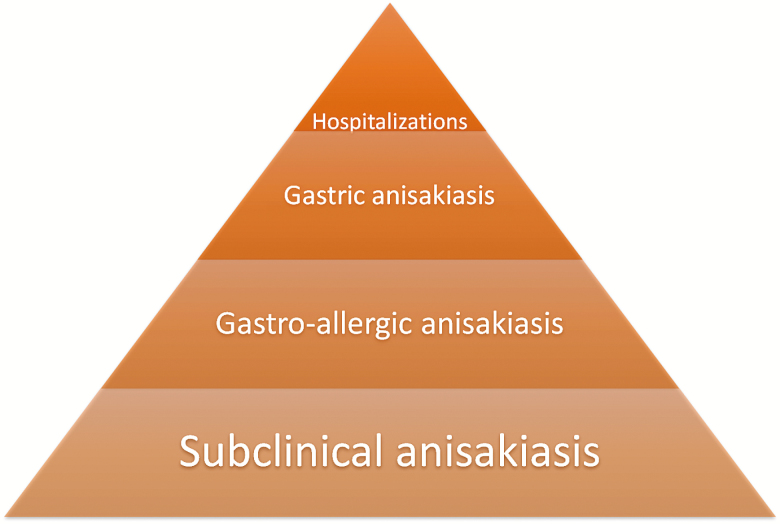
Relative anisakidosis incidence in its different clinical and subclinical forms. Our data show a hospitalization rate of 2.93 in Spain and 9.17 in Madrid per 1000000 population. In the same Madrid area, annual incidence due to gastroallergic anisakiasis has already been reported as 192 per 10000000 [7, 14]. An even higher rate of subclinical anisakiasis is suspected as indicated by high sensitization rate of the population in endemic areas [39]. Acute gastric anisakiasis is the most frequent clinical feature in Japan, but less so in Spain [35]. Only rare intestinal anisakiasis with surgical complications, concomitant disease, or, in some cases, anaphylaxis leads to hospitalization, as depicted by the top of the pyramid.

In a prospective study carried out in the Hospital La Paz, Madrid, in 1997 [14], only 1 of 96 patients with diagnosis of gastric or gastroallergic anisakidosis attended in the emergency room required hospitalization. In that year (1997), in Madrid province there were 10 anisakidosis-related hospitalizations for 5050000 inhabitants, yielding a similar hospitalization rate due to all *Anisakis*-associated diagnoses. In our study, the annual average of anisakidosis-related hospitalizations was 130. Knowing that only 1%–2% of anisakidosis require hospitalization [14], we estimate between 6370 and 12870 annual cases of anisakiasis requiring medical attention. Applying the calculated time trends over the study period (Figure 1), these numbers increase to 10383–20978 annual cases in the last few years. Figure 4 gives an estimation of incidence due to anisakidosis, where gastroallergic anisakidosis accounts for the majority of visible anisakidosis cases, an unknown number of acute gastric anisakidosis cases, and a small number of hospitalizations mainly due to known digestive and other complications.

## DISCUSSION

### Temporal and Geographical Trends in Anisakidosis-related Hospitalizations

We found a significant increasing trend in anisakidosis-related hospitalization rates in Spain during the whole study period. Surprisingly, 2 decades ago, anisakidosis was still relatively unknown in Western Europe [6, 15]. This incidence increases might have several explanations. First, we know that scientific and medical awareness as well as implementation of diagnostic tools has led to more frequent reporting of anisakidosis [16]. Several publications on a big series of anisakidosis appearing suddenly some 20 years ago point to awareness as a main factor of diagnostic trends [3, 9, 14, 17]. Second, the growing popularity of eating raw or uncooked seafood may have resulted in the proliferation of this parasitic infection [5]. Third, there may be a higher parasitism/infestation of fish related to current fishing and aquaculture practices, which elevates risk of anisakidosis in consumers [18, 19].

Anisakidosis outbreaks might have occurred during the study period. Local outbreaks have been very rarely described in the literature [20]. Unfortunately, the CMBD data do not allow us to assess if the peaks in our series represent aggregated cases.

Around a quarter of the cases occurred in the months of May and October. Monthly variations have been observed elsewhere [21, 22]. Seasonal fluctuations in the population dynamics of *Anisakis* species have been associated with seasonal fluctuations of sea temperature, current, and salinity, the impact of open and deeper waters, changes in the migration of aquatic mammals, the amounts of parasite eggs laid, and zooplankton availability [22, 23]. Other various topographic and hydrographical factors could also explain the seasonality of these nematodes [24], irrespective of other factors.

Relevant regional differences were found. Most affected communities were located in the northwest inland part of the country. These geographical differences may be due to differences in fish consumption habits combined with differences in the prevalence of infection among the fish consumed [25, 26]. In Italy, it was observed that anisakidosis was mostly transmitted by the ingestion of marinated anchovies in coastal areas and by fashionable foods (sushi, sashimi, etc) in inland areas [27]. Recent studies on fish parasitization in several Spanish marine areas indicate that the presence of larvae is very common, with relevant geographical differences: higher frequency in the Cantabrian Sea, with a prevalence of 50%, compared to the Atlantic Ocean (36%), while considerably lower in the Mediterranean Sea (6%) [3, 4, 22, 28–31]. Unfortunately, even if this geographical pattern helps us to understand our results, we still don’t know the origin of the fish products consumed in every region. Furthermore, as the main preventable anisakidosis risk factor is the final fish preparation method, regional differences in culinary habits may account mostly for epidemiologic data, as well as sensitization rates [26].

Failures in preventive actions constitute another possible explanation. Preventive measures are essential for anisakidosis control. Guidelines for risk reduction should cover all the food chain phases, from practices during the capture and subsequent handling, to technological treatments of processed products, to recommendations aimed at collective and consumer restoration. In European Union legislation, preventive measures for all the involved operators have been established [32]. In Spain, these regulations are complemented by the Royal Decree 1420/2006, which targets the prevention of parasitism by *Anisakis* species in products of the fisheries supplied to establishments that serve food [33]. However, the application of these national actions by regional health competent authorities may vary.

Our results partially differ from the estimations performed by Bao et al [7]. By using a quantitative risk assessment (QRA) model for the anchovy value chain, these authors estimated that 42% of cases occur in the Spanish communities of Andalusia and Madrid. In our study, the communities with the highest hospitalization rates were Madrid followed by Castilla-León and La Rioja, while Andalusia showed one of the lower rates. It is likely that the number of untreated anchovy meals consumed by Spaniards, which was the main parameter in this QRA, is insufficient for estimating the real disease burden.

### Characteristics of Anisakidosis-related Hospitalizations

Most hospitalized patients were aged 16–64 years. Sensitization to *Anisakis* has been reported to increase with age [34], and our own data are in accordance with most digestive and allergic diagnoses being more frequent at higher ages. This does not fit with classical age patterns in allergic disease, but with a higher risk of host immune response against invading parasites with age, as sensitization reflects previous contact with *Anisakis* irrespective of its clinical manifestation (digestive and/or allergic) [35].

The most frequent codiagnoses in anisakidosis-related hospitalizations belonged to the *ICD-9-CM* group diseases of the digestive system. Intestinal anisakidosis is considered a rare parasitic disease, difficult to diagnose due to the unspecificity of its symptoms and long time intervals [36]. In addition, intestinal anisakidosis may mimic several surgical conditions, including appendicitis, ileitis, diverticulitis, or inflammatory bowel disease. These are frequently primary diagnoses, which lead to surgery where anisakidosis is unexpectedly diagnosed [37, 38]. Awareness of these clinical manifestations’ relations with anisakidosis may facilitate its recognition and correct diagnosis, which is essential for the appropriate therapeutic approach.

Urticaria, angioneurotic edema, and anaphylactic reaction were recorded in <2.5% of anisakiasis-related hospitalizations. Part of these episodes could be due to side effects due to drug allergy. The higher proportions of allergic diagnoses are accompanying the parasitic episode of typical gastroallergic anisakidosis. This is further supported by 38 cases of anaphylaxis, which were registered in the first 2 diagnostic positions with only 2 digestive codiagnoses associated. Moreover, we cannot rule out that true anisakiasis with allergic symptoms could have been mistaken in the emergency room for drug allergy. With the inherent limitations of this database analysis, it thus seems that some of the patients were hospitalized due to gastroallergic anisakidosis with anaphylactic reaction, as has been characterized previously. Abdominal symptoms are mainly of slight nature and often absent. Our data are in agreement with anisakidosis patients hospitalized mainly due to abdominal complications of anisakidosis but only a very small proportion due to gastroallergic anisakidosis, which is self-limited with symptomatic medication in the vast majority of cases [14, 35]. In fact, previous reports have shown that appearance of allergy in the context of parasitism by *Anisakis* species leads to rapid expulsion of the larva from the stomach mucosa and that chronic anisakidosis complications do not have previous allergic episodes [39].

### Anisakidosis Burden in Spain

Hospitalization is very unusual in anisakidosis; only rare intestinal anisakidosis with concomitant surgical complications or, in some cases, anaphylaxis leads to hospitalization [7, 14, 15]. As depicted by the top of the pyramid in Figure 4, we should expect that the real figures largely exceed our hospitalization rates, especially when even a higher rate of subclinical anisakidosis is suspected, as indicated by high sensitization rate of the population in endemic areas [39]. Moreover, acute gastric anisakidosis is the most frequent clinical feature in Japan, but to a lesser extent in Spain [35]. According to our estimations, there could be around 10383 and 20978 annual cases in the last years in Spain, figures even higher than those calculated by Bao et al (approximately 7700–8320 cases per year) [7].

### Limitations and Conclusions

Several considerations should be taken into account when interpreting our findings. First, as previously discussed, we analyzed cases of anisakidosis requiring hospitalization, which is not equivalent to the true anisakidosis incidence. Second, the use of hospital records data for epidemiological consideration may be prone to imprecision due to the lack of relevant individual, clinical, and laboratory information. Moreover, the CMBD does not include information from all the private hospitals, but altogether the vast majority of anisakidosis cases are expected to be attended at the emergency room of public hospitals. Third, potential bias might have been introduced by sole reliance on *ICD-9-CM* codes.

In any case, our findings reported here have potential implications for public policy. We have demonstrated that anisakidosis is an emergent zoonosis in Spain. There is a need for a common national (an also international) strategy on data collection, monitoring, and reporting, which would facilitate a more accurate picture and strategic control measures design. Improving human and animal anisakidosis surveillance will be useful, both in gaining extended disease knowledge and reducing morbidity and related costs.

Finally, with our results, we have aimed at relieving the lack of official epidemiological data, but we also expect to have contributed to generate hypotheses that will be worthy to be explored in further investigations.

## Supplementary Data

Supplementary materials are available at *Clinical Infectious Diseases* online. Consisting of data provided by the authors to benefit the reader, the posted materials are not copyedited and are the sole responsibility of the authors, so questions or comments should be addressed to the corresponding author.

ciy853_suppl_Supplementary_Table_1

ciy853_suppl_Supplementary_Table_2

## Notes

***Acknowledgments.*** The authors thank the General Subdirection of the Institute for Health Information, Spanish Ministry of Health, Social Services, and Equality for providing the information on which this study is based. The corresponding author’s center belongs to the Network Biomedical Research on Tropical Diseases (RICET).

***Financial support.*** This work was supported by the European Regional Development Fund from the European Commission (contract number RD12/0018/0001).

***Potential conflicts of interest.*** All authors: No potential conflicts of interest. All authors have submitted the ICMJE Form for Disclosure of Potential Conflicts of Interest. Conflicts that the editors consider relevant to the content of the manuscript have been disclosed.
