# Supplementary material for: Epidemiological Scenario of Anisakidosis in Spain Based on Associated Hospitalizations: The Tip of the Iceberg
Source: Clin Infect Dis. 2018 Oct 3;69(1):69–76. doi: 10.1093/cid/ciy853 (PMC6579956; doi:10.1093/cid/ciy853)
Supplement: ciy853_suppl_Supplementary_Table_1 [file ciy853_suppl_supplementary_table_1.docx]

Supplementary Table 1. Mean anisakidosis hospitalizations rates per 1,000,000 population by autonomous community, 1997-2015, Spain.

| **Autonomous community** | **Population average** | **Average number of cases** | **Average rate*1,000,000** |
| --- | --- | --- | --- |
| **Andalusia** | 7,833,241 | 12 | 1.47 |
| **Aragon** | 1,273,267 | 4 | 3.27 |
| **Asturias** | 1,067,943 | 1 | 0.69 |
| **Balearic Islands** | 964,313 | 0 | 0.11 |
| **Canary Islands** | 1,880,374 | 0 | 0.08 |
| **Cantabria** | 561,676 | 0 | 0.56 |
| **Castilla-Leon** | 2,500,620 | 22 | 8.99 |
| **Castilla-La Mancha** | 1,918,266 | 9 | 4.53 |
| **Catalonia** | 6,920,268 | 6 | 0.92 |
| **Valencia** | 4,588,677 | 3 | 0.72 |
| **Extremadura** | 1,078,765 | 1 | 0.54 |
| **Galicia** | 2,733,007 | 0 | 0.12 |
| **Madrid** | 5,902,027 | 54 | 9.17 |
| **Murcia** | 1,325,206 | 1 | 0.79 |
| **Navarra** | 593,263 | 4 | 6.39 |
| **Basque Country** | 2,131,071 | 10 | 4.62 |
| **Rioja** | 298,479 | 2 | 8.29 |
| **Ceuta** | 75,588 | 0 | 0.00 |
| **Melilla** | 71,072 | 0 | 0.74 |
| **Total** | 43,983,069 | **130** | 2.96 |
