# Supplementary material for: Epidemiological Scenario of Anisakidosis in Spain Based on Associated Hospitalizations: The Tip of the Iceberg
Source: Clin Infect Dis. 2018 Oct 3;69(1):69–76. doi: 10.1093/cid/ciy853 (PMC6579956; doi:10.1093/cid/ciy853)
Supplement: ciy853_suppl_Supplementary_Table_2 [file ciy853_suppl_supplementary_table_2.docx]

Supplementary Table 2. Frequent co-diagnoses in anisakidosis related hospitalizations by sex, Spain, 1997-2015.

| **Co-diagnoses** | **ICD-9 codes** | **Total Population** | | **Males (n=1,318)** | | **Females (n=1,153)** | | **p value** |
| --- | --- | --- | --- | --- | --- | --- | --- | --- |
|  |  | **n** | **%** | **n** | **%** | **n** | **%** |  |
| **Most frequent digestive co-diagnoses** | | | | | | | | |
| Intestinal obstruction without mention of hernia | 560 | 312 | 12.63 | 210 | 15.93 | 102 | 8.85 | <0.01 |
| Gastritis and duodenitis | 535 | 192 | 7.77 | 91 | 6.90 | 101 | 8.76 | 0.086 |
| Other and unspecified noninfectious gastroenteritis and colitis | 558 | 188 | 7.61 | 89 | 6.75 | 99 | 8.59 | 0.086 |
| Regional enteritis | 555 | 89 | 3.60 | 47 | 3.57 | 42 | 3.64 | 0.919 |
| Cholelithiasis | 574 | 79 | 3.20 | 38 | 2.88 | 41 | 3.56 | 0.343 |
| Functional digestive disorders not elsewhere classified | 564 | 73 | 2.95 | 35 | 2.66 | 38 | 3.30 | 0.348 |
| **Most frequent allergy related co-diagnoses** | | | | | | | | |
| Other anaphylactic reaction. Anaphylactic reaction due to unspecified food | 995.0. 995.6 | 60 | 2.43 | 33 | 2.50 | 27 | 2.34 | 0.794 |
| Urticaria | 708 | 55 | 2.23 | 26 | 1.97 | 29 | 2.52 | 0.413 |
| Angioneurotic edema. not elsewhere classified | 995.1 | 29 | 1.17 | 14 | 1.06 | 15 | 1.30 | 0.582 |
|  |  |  |  |  |  |  |  |  |
| **Other frequent co-diagnoses** | | | | | | | | |
| Other symptoms involving abdomen and pelvis | 789 | 197 | 7.97 | 105 | 7.97 | 92 | 7.98 | 0.991 |
| Cardiac dysrhythmias | 427 | 138 | 5.58 | 76 | 5.77 | 62 | 5.38 | 0.674 |
| Asthma | 493 | 108 | 4.37 | 39 | 2.96 | 69 | 5.98 | <0.01 |
| Obstructive chronic bronchitis with (acute) exacerbation | 491.21 | 36 | 1.46 | 32 | 2.43 | 4 | 0.35 | <0.01 |
